# New Insights into the Cosmetic Behaviour of Bearded Vultures: Ferruginous Springs Are Shared Sequentially

**DOI:** 10.3390/ani13152409

**Published:** 2023-07-26

**Authors:** Antoni Margalida, Ivan Almirall, Juan J. Negro

**Affiliations:** 1 Pyrenean Institute of Ecology (CSIC), 22700 Jaca, Spain; 2 Institute for Game and Wildlife Research IREC (CSIC-UCLM-JCCM), 13071 Ciudad Real, Spain; 3 Cos d’Agents Rurals, Àrea Bàsica del Pallars Jussà, 25620 Tremp, Spain; ivan.almirall@gencat.cat; 4 Department of Evolutionary Ecology, Estación Biológica de Doñana (CSIC), 41092 Sevilla, Spain; negro@ebd.csic.es

**Keywords:** camera-trap, cosmetic soils, animal signals, *Gypaetus barbatus*, quality foragers, signalling status

## Abstract

**Simple Summary:**

The adventitious nature of the rufous colour of bearded vultures was first suggested in the XIXth century, and proved conclusively almost a century later. However, for more than 20 years, no advances about this mysterious behaviour have been made, and observational studies are needed to discover its function. Here, with the help of camera-traps and GPS transmitters we provide new observations about the regular use of a ferruginous spring situated in the Spanish Pyrenees by the species, providing new insights on the behavioural ecology of bearded vultures.

**Abstract:**

Different hypotheses have been proposed to explain the function of cosmetic behaviour of bearded vultures, being the signalling individual dominance status the most accepted. However, no advances have been made in understanding this mysterious behaviour, in part due to the secrecy of this species. With the help of camera traps and GPS devices we monitored the use of a ferruginous spring in the Pyrenees (Spain) providing new insights into this aspect of their behavioural ecology. Most of the visits (93.5%) involved a single bearded vulture and bathing behaviour only occurred when a single individual was present, confirming their secretive behaviour. A total of 50% of individuals that visited the site were non-adults, suggesting that cosmetic coloration functions as an attenuating signal that may also benefit subordinate individuals. Future studies with the help of new technologies could help to disentangle some questions about the real function of cosmetic coloration and their social relevance.

## 1. Introduction

An intriguing behaviour of bearded vultures (*Gypaetus barbatus*) is their habit of deliberately staining their feathers with red soil bathing [1]. Such active application of adventitious (external) pigments by a bird to its plumage has been termed cosmetic coloration [2]. The bright red-orange colours on the neck, head and ventral parts of bearded vultures are due to the iron oxide particles found in ochre in the applied soil [3,4,5,6,7]. Hominins, including *Homo neanderthalensis* and *H. sapiens*, have stained their bodies with ochre for more than 200,000 years [8], possibly in behavioural imitation, or biomimetism, of bearded vultures [6], whose evolution pre-dates that of humans [9], and which are distributed in Africa and Eurasia, coinciding with the cradle of the human lineage and its subsequent expansions out of Africa. In addition, hominins and vultures have shared the same habitats (i.e., rocky outcrops and caves) and foraging practices (i.e., carcasses of large animals) for millennia [10].

The adventitious nature of the rufous colour of bearded vultures was first suggested in the 19th century [11], and proved conclusively almost a century later [12]. Different hypotheses have been proposed to explain the function of cosmetic behaviour, including: (1) physical protection against feather abrasion [3]; (2) signalling individual dominance status [1]; (3) antiparasitic and antibacterial plumage protection [3,13,14]; (4) the provision of antioxidant properties to developing embryos via the transfer of iron oxides through the eggshell [14]; and (5) the action of stained plumage as an attenuating signal [15]. This last and most recently proposed hypothesis suggests that birds with feather imperfections may benefit from concealing them with ochre. Low-quality individuals (e.g., subordinate individuals with poorly maintained plumage) would benefit from staining, while high-quality individuals would pay a social (breeding) cost for hiding their immaculate plumage [16]. While signalling individual dominance status (hypothesis 2) has received wide support [1,17,18,19], the other hypotheses have been rejected through observational and experimental approaches (hypotheses 1 and 3) or remain untested (hypotheses 4 and 5) [15,17,19,20]. However, no advances have been made in understanding this mysterious behaviour during the last two decades, and observational studies are needed to discover its function.

The information regarding the use of such resources and the exact function of cosmetic behaviour is still tentative due to the extreme rarity of field data [21,22,23] for bearded vultures and [24] for Egyptian vultures. This is in part due to this species secretive behaviour [4,13] and also the difficulty of obtaining high quality data on this uncommon threatened species in their mountainous habitats. In this paper, we provide new observations on the regular use by bearded vultures of a ferruginous spring situated in the Spanish Pyrenees using camera-traps and GPS transmitters, to provide new insights into this aspect of their behavioural ecology.

## 2. Methods

**Figure 1:**
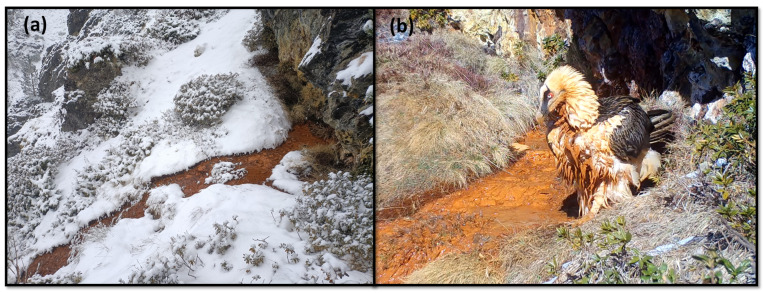
A ferruginous spring in winter. (**a**) The regular presence of water allows bearded vultures to visit the spring during the entire year. (**b**) An adult bearded vulture after having taken a bath.

The study was carried out in the Pyrenees (NE Spain). We intermittently monitored a ferruginous spring in the Catalonian Pyrenees using a Recon Force Advantage camera trap (Browning Trail Cameras, Birmingham, AL, USA; Figure 1) between October 2021 and February 2023. To be inconspicuous in the landscape, the camera was fixed and camouflaged with natural elements (branches, leaves) present in the same site. Cameras were programmed to take consecutive videos (30 s) when movement was detected. For each bearded vulture visit recorded, we obtained the data and hour (solar time), number of individuals present, estimating the age, behaviour (perched, bathing, drinking), time of presence (in minutes). The individuals that visited the ferruginous spring were identified based on the individual characteristics of their plumage and the individual peculiarities of the shapes of their pectoral bands and crowns. Age-classes categories were established according to the plumage characteristics [25]: juveniles (until two years old), immatures (three years old), subadults (four-five years old) and adults (over six years old). In parallel, between 2006 and 2022 we monitored 31 bearded vultures with solar-powered GPS/GSM transmitters. A total of 20 individuals were monitored with Microwave Telemetry (Microwave Telemetry Inc., Columbia, MD, USA) and 11 with Ornitela transmitters (Ornitela https://www.ornitela.com, accessed on 15 July 2023), attached using a Teflon backpack harness. The total weight of the transmitters and rings represented less than 3% of the body weight of the individuals. We checked the presence of these tracked individuals during the period of monitoring of the ferruginous spring with the camera trap (October 2021–February 2023) to assess the frequency of visits. During this period only 13 (41.9%) of the 31 individuals tracked with GPS transmitted information (due to mortality or battery loss). Values are presented as means ± sd.

## 3. Results

**Figure 2:**
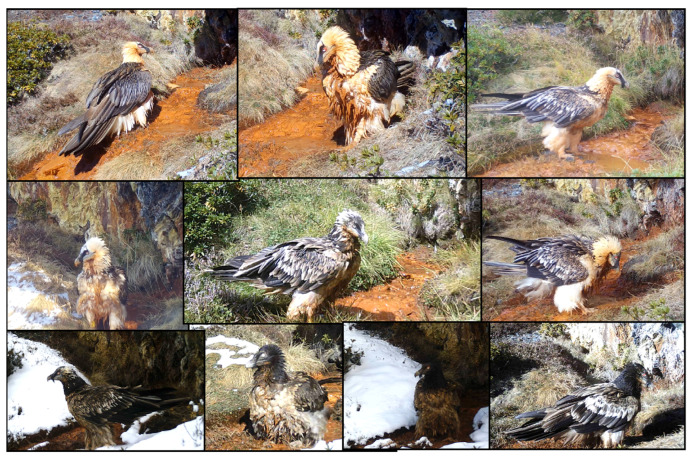
Bearded vultures of different age-classes visiting the ferruginous spring at different times of the year with and without snow around the site. From top to bottom and left to right, adult individuals (pictures 1 and 2), subadult (pictures 3, 4, 5, 6 and 7), immature (pictures 8 and 9) and juvenile (picture 10).

At least 24 individuals from different age-classes visited the spring (diameter: 75 cm; depth: 2.8 cm). The camera, situated 4 m away from the spring, recorded vultures every six days on average (33 different days out of 207 days operation). Individual plumage characteristics allowed the identification of four juveniles (two years old), three immatures (three years old), six subadults (four–five years old) (near adult individual with a non-completed moult, still showing some feathers from the subadult plumage, five years old) and ten adults (over six years old) (Figure 2).

**Figure 3:**
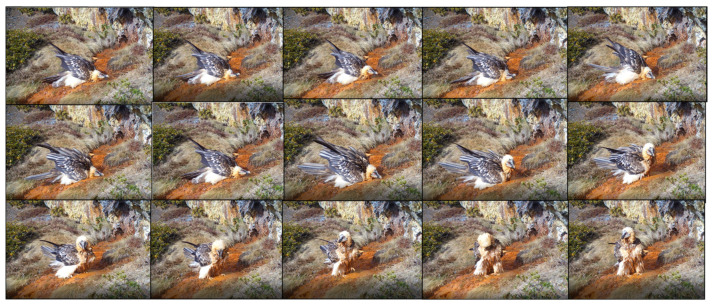
Sequence showing a subadult bearded vulture taking a bath.

Bearded vultures mainly visited the spring during the middle of the day, with bathing visits concentrated between 09:00 and 16:00. Most of the visits (93.5%, *n* = 31) involved a single individual. On two occasions, two individuals were present at the same time (one adult and one juvenile), and on another, three individuals were present (two adults and one juvenile). Bathing behaviour (*n* = 15) only occurred when a single individual was present (Figure 3). Of the 24 individuals that visited the site, 15 used the spring for bathing (eight adults, two juveniles, one immature, and four subadults). The rest (*n* = 9) just drank water and snow, or simply perched on the ground before immediately leaving. The average number of visits per individual was 1.43 ± 1.05 (range 1–5), and 80.9% only visited the site once. The mean duration of stays at the spring was 10.03 ± 11.69 min (*n* = 36).

Information obtained from a single GPS-tracked individual (a subadult) that was photographed at the spring on the 12th and 13th April 2022, indicated that this same bird visited the spring on five other occasions. Monitoring in 2023 also showed this bird visiting the site once in February, indicating that this four-year-old visited the spring on seven occasions during a full year. Thus, this implies that 7.7% of the individuals with GPS devices visited the site.

## 4. Discussion

Based on the literature review by [2] and recent observations of Egyptian vultures [24], the use of cosmetic substances has been described in 29 bird species belonging to 13 families. Our observations are the first to document visits by individuals of all age-classes to a given ferruginous spring, constituting a significant part (2.5%) of the entire Pyrenean mountain range population (estimated at 1026 individuals; [26]). The only previous information available in the literature [23] showed that during 1483 days of monitoring a ferruginous spring in the French Pyrenees, bearded vultures were present on 112 occasions (7.55%) and ten individuals were identified (nine adults over six years old and one five-year-old).

Ferruginous waters are not ubiquitous in the landscape, so that potential bearded vulture bathing sites are geographically sporadic [1,4]. In addition, due to their secretive behaviour [4,13] and the mountainous landscapes inhabited by bearded vultures, the observations available of this behaviour are very limited. For example, during the long-term study of the species carried out in the Pyrenees by one of us (33 years), we only observed direct or indirect bathing behaviour on two occasions, identifying three areas with ferruginous sources used by bearded vultures. In addition, probably only some individual vultures have ready access to cosmetic soils. The variability of the intensity of the orange in the vultures’ plumage, and the presence of white or very pale individuals, shows that many birds never, or only occasionally, have access to cosmetic soils. Finding sources of the cosmetic iron oxides is therefore costly in terms of time and search effort, adding to the more general costs of preening [1,2]. This accords with the hypothesis of [27] that visual signals need to be costly to demonstrate significant investment and be honest and thus reliable for receivers. Our observations show that this ferruginous spring is an important source used: (1) by adult individuals to acquire the orange coloration to signal their ability to find restricted resources to conspecifics (so demonstrating quality as foragers); and (2) also by immature individuals, which have brown plumage against which the orange coloration is not readily seen by a human observer. The possibility that cosmetic coloration functions as an attenuating signal (the opposite of an amplifier) that could benefit low-quality and/or subordinate individuals merits future study, especially since it has been shown to be a theoretically possible evolutionary stable strategy [28].

Camera-traps and GPS devices can provide detailed information about the use of ferruginous springs and their social importance. Our observations showed that the monitored spring was visited by several individuals of different age-classes. GPS accelerometer techniques and monitoring of a variety of sources of cosmetic pigments could improve our understanding of the social signalling relevance and functional benefits derived from the use of iron oxide pigments [29]. For example, in captivity, the intensity of the ochre coloration generally correlates with the age and sex of the bird, with older individuals and females being more intensely pigmented [13]. In wild individuals, females (the dominant sex) also show more intense coloration than males and, in the case of polyandrous trios, alpha males are more heavily stained than beta males [1,18]. Thus, a more intense use of pigmentation sites by females could explain sexual differences in coloration. Our observations suggest that the same individual may visit a single site up to seven times over the course of a year, although of course other ferruginous springs might be visited in other areas. In fact, territorial adults can exploit home ranges (Kernel 90) of about 63 km^2^, while non-territorial birds can use areas of around 11,400 km^2^ [30]. Thus, territorial adults can be more limited visiting a few ferruginous sites near their breeding territories while non-territorial ones (i.e., juvenile, immature and subadult individuals) exploring large areas could have access to different sources. New technologies provide excellent monitoring and field experimental opportunities using free-ranging individuals. The identification of areas containing ferruginous sources together with use of GPS transmitters and camera-traps will improve our knowledge of cosmetic behaviour and provide important insights into its function. On the other hand, this study is the first to show the importance that ferruginous sources have for this threatened species due to the high number of individuals that can visit a single site. As occurs with breeding sites, the protection of ferruginous springs should be considered by managers and policy-makers as a priority conservation area to avoid disturbance. Advancing the socio-ecological understanding of the social life of vultures seems critical to harmonize their conservation in a rapidly changing world [31]. Accordingly, a next step by managers and policy-makers should be the identification of used and/or suitable ferruginous sites in the distribution area of the species, in order to apply protection and regulatory guidelines to these sites.

## 5. Conclusions

The information of the use of cosmetics by bearded vultures to improve our knowledge about the functionality of this behaviour is still hypothetical due to the extreme rarity of field data. This is in part due to their secretive behaviour and the difficulty to obtain high data quality in mountainous landscapes inhabited by this threatened species. Here, with the help of camera-traps and GPS transmitters we provide new observations about the sequential use by different age-class individuals of a ferruginous spring situated in the Spanish Pyrenees. The possibility that cosmetic coloration functions as an attenuating signal (the opposite of an amplifier) that could benefit low-quality and/or subordinate individuals (50% of the individuals observed) merits future study. In addition, the socio-ecological understanding of the social life of vultures seems critical to harmonize their conservation in a rapidly changing world. The protection of ferruginous springs should have relevance from a conservation perspective to avoid disturbance on these sensitive sites. As a result, the identification of used and/or suitable ferruginous sites in the distribution area of the species to apply protection and regulatory guidelines to these sites, could be an important management and conservation action for this threatened species.

## Data Availability

The data presented in this study are available in this article and from the corresponding author upon reasonable request.
